# Acute effect of statins on vascular reactivity in maternal and placental arteries from pregnancies complicated by preeclampsia

**DOI:** 10.3389/fphys.2025.1575128

**Published:** 2025-06-24

**Authors:** Chinedu Agwu, Jenny Myers, Mark Wareing, Mark Dilworth

**Affiliations:** ^1^ Maternal and Fetal Health Research Centre, Division of Developmental Biology and Medicine, Faculty of Biology, Medicine and Health, University of Manchester, Manchester, United Kingdom; ^2^ Saint Mary’s Hospital, Manchester University NHS Foundation Trust, Manchester, United Kingdom; ^3^ Manchester Academic Health Science Centre, Manchester, United Kingdom

**Keywords:** preeclampsia, statins, pregnancy, vascular function, therapeutics, pitavastatin and pravastatin

## Abstract

**Introduction:**

This study aimed to investigate the acute effects of statins on maternal and fetoplacental vascular reactivity in vessels from pregnancies affected by pre-eclampsia (PE), a leading cause of maternal and fetal morbidity and mortality. Statins have been proposed as a candidate therapy due to their pleiotropic effects but evidence of statins’ ability to ameliorate the observed endothelial dysfunction in PE is lacking.

**Methods:**

Human chorionic plate arteries (CPAs) and omental arteries (OAs) from normal and PE pregnancies were mounted on a wire myograph. Contraction was assessed with KPSS and the thromboxane mimetic U46619. Arteries were incubated for 2 h with 1 µM or 10 µM pravastatin, pitavastatin or simvastatin (pitavastatin only in OAs). U46619 dose–response curves were repeated or dose-response curves with NO-donor SNP or endothelium-dependent bradykinin (BK) performed following U46619 pre-constriction.

**Results:**

CPAs from normal and PE pregnancies showed similar responses following exposure to the vasoconstrictive agent U46619 and the relaxatory agent SNP. Short-term exposure to pravastatin, simvastatin and pitavastatin did not cause detrimental effects on CPA reactivity. Acute exposure of OAs from PE pregnancies to pitavastatin (1 µM) did not reduce U46619-mediated contraction or enhance BK-mediated relaxation of vessels although in this study *ex vivo* endothelial function of OAs from PE pregnancies was not different to those in normotensive pregnancy pre incubation.

**Discussion:**

In conclusion, this study did not demonstrate an effect on vascular reactivity of maternal systemic or fetoplacental arteries following acute treatment of statins. Future studies investigating the effect of longer-term statin exposure on maternal and fetoplacental vascular reactivity may help towards treatment strategies for vascular dysfunction in PE-affected patients.

## 1 Introduction

Preeclampsia (PE) is defined by hypertension at ≥ 20 weeks gestation and proteinuria or evidence of placental, renal, hepatic, or hematological dysfunction (Khan et al., 2020). PE affects 4%–5% of pregnancies and is a major contributor to maternal and fetal morbidity and mortality (Phipps et al., 2019), including increased risk of preterm birth and fetal growth restriction (FGR) (Haddad et al., 2004; Madazli et al., 2014; Rezk et al., 2015). Additionally, women diagnosed with PE have an increased risk of cardiovascular disease (CVD) later in life (Kuklina et al., 2009; Chen et al., 2014; Breetveld et al., 2014; Wu et al., 2017; Leon et al., 2019; Ormesher et al., 2022). Meta-analyses indicate an estimated 3.7-fold increased risk for hypertension and 1.8-fold for stroke in women previously diagnosed with preeclampsia (Bellamy et al., 2007).

PE is associated with placental dysfunction and placental ischemia (Bakrania et al., 2020; Chappell et al., 2021), and severe early-onset cases of PE are linked to inadequate spiral artery remodeling (Labarrere et al., 2017; Staff et al., 2020). In addition, PE is associated with an angiogenic imbalance in the maternal circulation (Ahmad and Ahmed, 2004; Phipps et al., 2019; Kluivers et al., 2023), ultimately resulting in maternal endothelial dysfunction (Maynard et al., 2003; Levine et al., 2004; Possomato-Vieira and Khalil, 2016; Tomimatsu et al., 2019; McElwain et al., 2020).

There are no effective treatments for PE. Clinical management following diagnosis currently focuses on the use of antihypertensive medications such as labetalol and nifedipine (NICE, 2023) and increased fetal monitoring. The only current treatment option for clinicians is delivery, often preterm, of the fetus and placenta. While this intervention halts the clinical progression of PE, there remains an urgent need for effective maternal treatment as an alternative to preterm birth (Berzan et al., 2014; Rana et al., 2019; Maayeh and Costantine, 2020).

Several drugs have been suggested for the treatment of PE, including statins, which are HMG-CoA inhibitors primarily used to reduce cholesterol levels (Phipps et al., 2019). Statin use in pregnancy was previously contraindicated, but progress has been made now that the FDA has requested removal of the “Pregnancy Category X” label for statins (US Food and Drug Administration, 2024). This was mainly due to previous results from systematic reviews and cohort studies linking increased rates of fetal abnormalities with statin exposure (Bateman et al., 2015; Edison and Muenke, 2004; Pollack et al., 2005; Taguchi et al., 2008; Winterfield et al., 2013). Although these studies are limited in their size and scope, current guidance does not recommend statin use during pregnancy.

Several clinical and preclinical studies using pravastatin have been performed, demonstrating lower rates of preeclampsia and improvement in the sFlt-1/PlGF ratio, yet showing little or no definitive evidence of benefit. Many of the clinical trials were underpowered (Ahmed, 2011; Brownfoot et al., 2016; Costantine et al., 2016; Costantine et al., 2021; Döbert et al., 2021; Mendoza et al., 2021). These studies have shown no evidence of detrimental effects of statins on the placenta, but there are limited *in vivo* data (Zarek et al.,2013).

Currently, there is a lack of knowledge on the effect of statins on vascular reactivity in either the maternal systemic arteries or the placental arteries. Various statins have been explored to assess the biological plausibility of statins for treatment in preeclampsia. These include lovastatin (Minsker et al., 1983), simvastatin (Cudmore et al., 2007; Rossoni et al., 2011), and pravastatin (Ahmed et al., 2010; Costantine et al., 2011; Kumasawa et al., 2010), although Rossoni and colleagues only investigated the acute effects of simvastatin on isolated mesenteric resistance arteries in male Wistar rats. Three statins were chosen for the current study: pravastatin, pitavastatin, and simvastatin. These were used to assess chorionic plate artery (CPA) reactivity, while pitavastatin alone was used for omental arteries (OAs) from pregnancies complicated by PE.

The different metabolic states of these three statins made them useful for comparison of pleiotropic effects on vascular function. Pitavastatin has high bioavailability in the bloodstream due to its avoidance of the first-pass metabolism route in the liver. Pitavastatin has never been used in pregnancy studies previously and is therefore an exciting therapeutic candidate to explore. In the current study, OAs and CPAs were used as models of the maternal systemic and fetoplacental vasculature, respectively. CPAs are resistance arteries, and they, along with other fetoplacental small arteries, play an important role in regulating vascular tone in the fetoplacental circulation (Mulvany and Aalkjaer, 1990).

We thus hypothesized that short-term (2 h) exposure to statins would improve the vascular reactivity of omental arteries from pregnancies complicated by preeclampsia and have no detrimental effects on fetoplacental CPA function. The aims of the study were 1) to assess whether vascular reactivity of OAs or CPAs was altered in PE *versus* normal pregnancies and 2) to assess whether short-term statin exposure affects vascular function in OAs and CPAs from both uncomplicated pregnancies and those complicated by PE.

## 2 Materials and methods

### 2.1 Ethical approval

All work herein was carried out following Research Ethics Committee approval (REC number 15/NW/0829). Studies were carried out in accordance with the guidelines of the Declaration of Helsinki. Written informed consent was obtained from all women prior to the collection of placental and omental samples.

### 2.2 Inclusion/exclusion criteria

Placental samples were taken from women with a normal pregnancy (normal defined as an uncomplicated pregnancy birthing ≥37 weeks with an appropriately grown fetus; N = 21) or a pregnancy complicated by PE (PE as defined by ISSHP guidelines (Brown et al., 2018; Duhig et al., 2019); N = 14). Following a Cesarean section, omental samples were taken from women with a normal pregnancy (N = 34) or a pregnancy complicated by PE (N = 9).

To control for women in the obese category, a BMI cut-off of <35 kg/m^2^ was used, and to control for women of advanced maternal age (aged 35 years and above), a cut-off of <40 years was utilized for uncomplicated pregnancies. Normotensive women were excluded if they had a BMI ≥35 kg/m^2^ or age ≥40 years, or they presented with maternal diabetes, multiple pregnancy, hyperthyroidism, or had other existing medical conditions or complications of pregnancy. Women with PE were excluded if they presented with diabetes, hyperthyroidism, multiple pregnancy, or if they had sexually transmitted diseases. Samples were considered “normal” and included if the individualized birthweight ratio (IBR) was between the 10th and the 100th centiles. IBR was calculated using the UK BulkCentileCalculator GROW 8.04 (Perinatal, 2019). Large-for-gestational age fetuses (>90th centile (Pasupathy et al., 2012)) were included where maternal characteristics were normal.

Unless otherwise stated, all chemicals and statins were obtained from Sigma–Aldrich (Poole, Dorset, United Kingdom) or BDH (Poole, Dorset, United Kingdom).

### 2.3 Sample collection

Omental biopsies ∼2 × 2 cm in size were excised from the greater omentum in the abdomen of women at the time of Cesarean section from normal pregnancies. Following collection, fat and scar tissue were removed, and OAs (138–521 µM) were isolated from the omental biopsy and placed directly into ice-cold physiologic salt solution (PSS). Resistance arteries were identified and mounted under a microscope onto a DMT multi-chamber 620 wire myograph using small dissecting scissors and forceps. For placentae, biopsies were taken and placed into ice-cold PSS, and small chorionic plate arteries (CPAs) were identified under the microscope and dissected free from surrounding connective tissue (112–501 µM) within 20 min of delivery, before being mounted onto a wire myograph.

### 2.4 Wire myography

Short (∼2 mm long) sections of omental arteries (OAs) and CPAs were mounted onto a Multi Myograph System 620M (Wareing et al., 2002). Initially, the bath contained 6 mL of PSS (in mmol 1^–1^; 127.76 NaCl, 25 NaHCO_3_, 4.69 KCl, 2.4 MgSO_4_, 1.6 CaCl_2_, 1.18 KH_2_PO_4_, 6.05 glucose, 0.034 EDTA; pH 7.4), warmed to 37°C, and gassed with 20% air/5% CO_2_ (OA) and 5% air/5% CO_2_ (CPA). Mounted OAs were normalized to 0.9L_13.3_ kPa while CPAs were normalized to 0.9L_5.1_ kPa following the method described in detail elsewhere (Wareing et al., 2002).

### 2.5 Contraction/relaxation responses for omental arteries/CPAs

Following equilibration, contractile viability was assessed with 2x high potassium PSS (KPSS; 120 mM KCl, equimolar substitution for NaCl) exposures with 10–20-min intervals in between. This was followed by concentration–response constriction curves to U46619 for OA/CPA (incremental doses of 10^–10^–10^–5.7^ M; 2 min intervals). Vessels (OAs) were washed to baseline tension with PSS and then incubated for 2 h with either 1 µM or 10 µM pitavastatin, 1 µM simvastatin, or 1 µM or 10 µM pravastatin (CPAs), with an appropriate control (1 µM DMSO or water) running in parallel. Following incubation, constriction to U46619/relaxation to bradykinin (BK; 10^–10^–10^–5^ M) was performed for OAs, and constriction to U44619/relaxation to sodium nitroprusside [SNP; (10^–10^–10^–5^ M)] was performed for CPAs. U44619-mediated contraction and basal tone pre- and post-statin were assessed to confirm if any statin-related effects were observed.

### 2.6 Statistical analyses

The Kolmogorov–Smirnov test was used to assess whether the data fitted a Gaussian distribution. This is used for datasets where only one comparison was made; for example, basal tone pre- and post-statin incubation and KPSS contraction pre- and post-statin incubation. Demographics were compared using the Mann–Whitney U test and Fisher’s exact test for categorical data, that is, smoking, parity, and sex of fetus, while the chi-squared test was used for ethnicity. U46619-induced constriction and SNP/bradykinin-induced relaxation of OAs and CPAs from women with normal and pathological pregnancies post-statin incubation were assessed using repeated-measures two-way ANOVA. Data are expressed as mean ± S.E.M, median ± SD, or median (range) depending on the outcome of the normality tests. The number of observations (n = arteries from N patients) is given in parentheses. Statistical significance was considered to be p < 0.05.

## 3 Results

### 3.1 Patient characteristics

Patient demographics for women donating placental and/or omental samples from NP and PE pregnancies are shown in Tables 1, 2. Large-for-gestational age (LGA) fetuses (>90th centile (Pasupathy et al., 2012) were included where maternal characteristics were normal. There were 7/34 LGA fetuses in the normotensive omental group only. Demographic clinical details for the two groups are shown in Tables 1, 2 (samples for placental and omental analyses are from different patients). Blood pressure was taken at booking (approximately 12 weeks of gestation) for both normotensive women and women who ultimately were diagnosed with PE; however, maximal blood pressure near delivery was collected for the PE group only. As expected, maximal recorded systolic (p < 0.01) and diastolic blood pressures (p < 0.05) were significantly higher in the PE group than in NP women (Mann–Whitney U test). Gestational age at delivery (p < 0.0001), birthweight (p < 0.0001), and IBR (p < 0.01, omental samples only) were significantly lower in the PE group than the NP group (Mann–Whitney U test). Furthermore, for women donating placental samples, a significant difference was seen with regard to parity (p < 0.0001) and a lower BW:PW ratio (p < 0.05) in the PE group. Smoking status, parity, and sex of the fetus were also different (Fisher’s exact test). Maternal age, BMI, and gravidity were not different between groups in those individuals donating omental and placental samples, while for omental samples, ethnicity was also significantly different between groups (p < 0.001).

**TABLE 1:** The maternal and pregnancy characteristics of women who agreed to the recruitment of omental samples are summarized below. For quantitative non-parametric data, information is presented as median and range. Statistical tests used for numerical data (Mann–Whitney U test) and categorical data (Fisher’s exact test or chi-squared test).

	Normotensive women (N = 34)	PE women (N = 9)	*p-*value
Maternal age (years)	**33** (18–39)	**36** (18–41)	0.778
Body mass index (kg/m^2^)	**26.6** (19.5–32.8)	**27.9** (18.3–35.6)	0.273
Birthweight (g)	**3447** (2660–4454)	**1667** (623–2658)	**** <0.0001
Individualized birth weight ratio	**50.6** (13.4–96.9)	**10.4** (0.4–31) **<3**rd centile = 4	** 0.003
Trimmed placental weight (g)	**514.4** (373.6–591.7)	**272.2** (57.6–560.3)	** 0.002
Birth weight: placental weight (BW:PW) ratio	**6.7** (5.0–7.7)	**6.1** (4.7–10.8)	0.195
Gestation (days)	**273** (259–285)	**236** (188–274)	**** <0.0001
Maternal systolic blood pressure (mmHg)			
Booking	**107** (90–124)	**110** (88–143)	** 0.001
Max	-	**150** (134–181)	- -
Maternal diastolic blood pressure (mmHg)			
Booking	**65** (52–80)	**68** (58–87)	* 0.011
Max	-	**99** (82–114)	-
Proteinuria (mg/mmol)	-	**181.5** (15–1181)	-
Parity			
Nulliparous	**20%**	**22%**	0.862
Gravidity	**3** (1–6)	**2** (1–5)	0.909
Ethnicity			
Caucasian	**57%**	**56%**	0.414
Afro-Caribbean	**6%**	**11%**	
Asian	**17%**	**11%**	
Other	**20%**	**22%**	
Smoking status			
Yes	**9%**	**0%**	** 0.0032
No	**91%**	**100%**	
**Sex of fetus**	**Female (37%)** **Male (63%)**	Female **(100%)** Male **(0%)**	**** <0.0001

**TABLE 2:** The maternal and pregnancy characteristics of women who agreed to the recruitment of placental samples are summarized below. For quantitative nonparametric data, information is presented as median and range. Statistical tests used for numerical data (Mann–Whitney U test) and categorical data (Fisher’s exact test or chi-squared test).

	Normotensive women (N = 21)	PE women (N = 14)	*p-*value
Maternal age (years)	**33** (23–47)	**31** (19–41)	0.175
Body mass index (kg/m^2^)	**23.4** (18.5–36.2)	**25.9** (20.8–35.2)	0.197
Birth weight (g)	**3395** (2282–3870)	**1670** (572–3750)	*** 0.0002
Individualized birth weight ratio	**56.7** (17.2–90.0)	**50** (0–94) **≥3**rd **to <10**th **centile** = 1 **<3**rd **centile** = 6	0.227
Trimmed placental weight (g)	**530.0** (374.4–624.3)	**297** (274.5–697.6)	*** 0.0008
Birth weight:placental weight (BW:PW) ratio	**6.1** (5.2–7.9)	**5.4** (2.7–8.0)	* 0.048
Gestation (days)	**273** (241–285)	**238** (195–277)	*** 0.0007
Maternal systolic blood pressure (mmHg)			
Booking	**106** (90–124)	**120** (100–143)	*** 0.0001
Max	-	**149** (134–181)	-
Maternal diastolic blood pressure (mmHg)			
Booking	**65** (52–78)	**76** (60–92)	** 0.003
Max	-	**109** (82–112)	-
Proteinuria (mg/mmol)	-	**241** (26–538)	-
Mode of delivery	**ELCS** (100%)	ELCS (38%) EMCS (47%) NVD (15%)	-
Parity			
Multiparous	**87%**	**62%**	**** <0.0001
Nulliparous	**13%**	**38%**	
Gravidity	**3** (1–6)	**2** (1–5)	0.295
Ethnicity			
Caucasian	**71%**	**38%**	**** <0.0001
Afro-Caribbean	**8%**	**8%**	
Asian	**8%**	**31%**	
Other	**13%**	**23%**	
Smoking status			
Yes	**8%**	**15%**	0.078
No	**92%**	**75%**	
Sex of the fetus	Female (48%) Male (52%)	Female (43%) Male (57%)	0.570

### 3.2 Assessment of vascular reactivity

The mean diameter of CPAs was not statistically different prior to treatment with statins between normal and PE pregnancies, and diameters were 314 µm [218–485] and 272 µm [173–414], respectively (*p =* 0.087). The mean diameter of OAs was also not statistically different prior to treatment with statins between normal and PE pregnancies; diameters were 324 µm [173–536] and 333 µm [239–466], respectively (p = 0.781).

### 3.3 Omental arteries

#### 3.3.1 Assessment of vascular reactivity in normal and PE pregnancies

**FIGURE 1:**
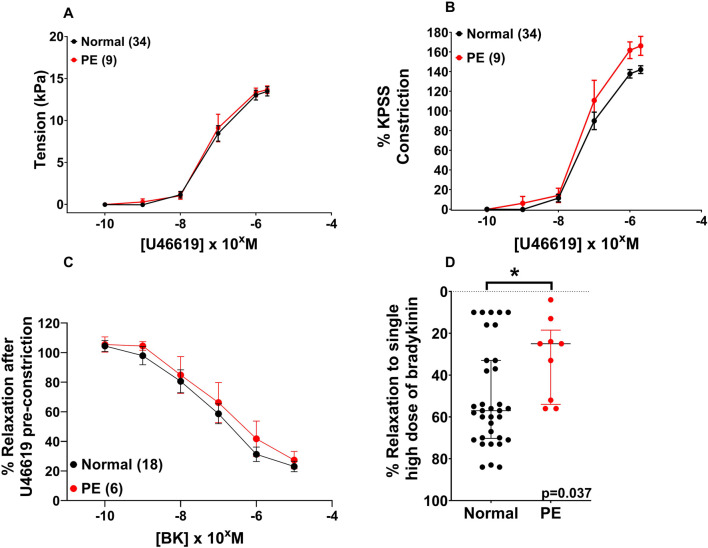
Assessment of vascular reactivity in OAs from normal and PE pregnancies. Assessment of U46619-induced contraction expressed as tension **(A)** or as %KPSS **(B)**. Relaxation to bradykinin post-incubation **(C)**. Compared to the endothelial function pre-incubation, there was a significant change in endothelial function between the NP and PE groups after relaxation with bradykinin **(D)**. Replicate vessels from the same pregnancy were averaged for each sample. Graphs A and B show the combined pre-incubation U46619 data for normal pregnancy (N = 34) from the pitavastatin (N = 18) and pravastatin (N = 16) experiments. Data are expressed as mean ± SEM; two-way ANOVA **(A–C)** and as median, IQR with Mann–Whitney test for graph **(D)**.

There was no significant difference in U46619-induced contraction between the NP and PE groups when expressed as tension (kPa) (Figure 1A, p = 0.557), or when U46619 data were expressed as %KPSS = high potassium physiological saline solution (Figure 1B, p = 0.188). When assessing endothelial function pre-incubation, there was significantly less relaxation to bradykinin in OAs from PE *versus* normal pregnancies (Figure 1D, p = 0.037). Following the 2-h incubation with DMSO, relaxation of OAs to BK was not different between normal or PE OAs (Figure 1C, p = 0.974). A high concentration of U46619 (10^–5^ M) was used to pre-constrict vessels prior to relaxation.

#### 3.3.2 Effect of statins on vasoconstriction of omental small arteries

**FIGURE 2:**
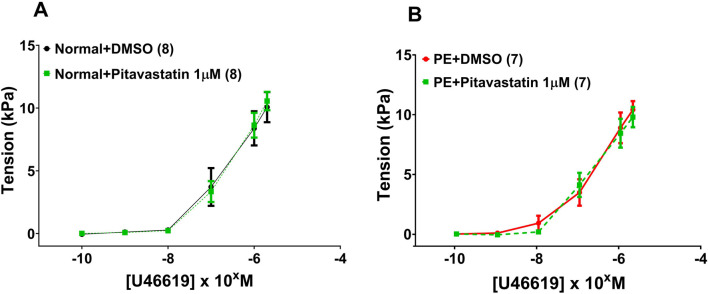
Effect of 2-h incubation with 1 µM pitavastatin on the contraction of OAs from normal pregnancies. Assessment of U46619-induced contraction expressed as tension (kPa) in normal pregnancy **(A)** and PE pregnancy **(B)**. There was no significant effect on the contraction of OAs. U46619 dose–response curves were compared using two-way ANOVA. Data are expressed as mean ± SEM; number of omental biopsies in parentheses.

Following a 2-h incubation with 1 µM pitavastatin, there was no significant effect on the contraction of OAs to U46619 from normal pregnancies (Figure 2A: DMSO *versus* pitavastatin, p = 0.980) or from PE pregnancies (Figure 2B: DMSO *versus* pitavastatin, p = 0.796).

#### 3.3.3 Effect of statins on vasodilatation of omental small arteries

**FIGURE 3:**
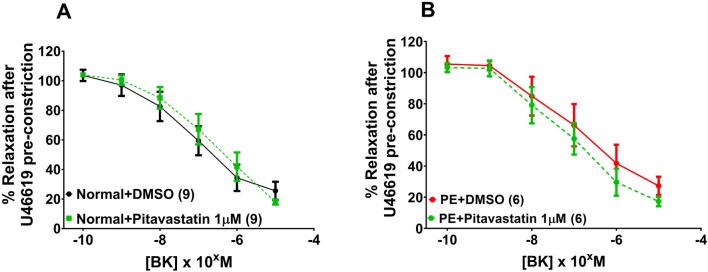
Effect of 2-h statin incubation on relaxation of OAs from normal pregnancies. Following a 2-hr incubation with pitavastatin at 1 µM, there was no significant effect on relaxation of OAs to BK in vessels from normal pregnancies **(A)** or from PE pregnancies **(B)**. BK dose–response curves were compared using two-way ANOVA. Data are expressed as mean ± SEM; number of omental biopsies in parentheses.

Following a 2-h incubation with 1 µM pitavastatin, there was no significant effect on relaxation of OAs to BK in vessels from normal pregnancies (Figure 3A: DMSO *versus* pitavastatin, p = 0.690) or from PE pregnancies (Figure 3B: DMSO *versus* pitavastatin, p = 0.963).

### 3.4 Chorionic plate arteries

#### 3.4.1 Assessment of CPA vascular reactivity in normal and PE pregnancies

**FIGURE 4:**
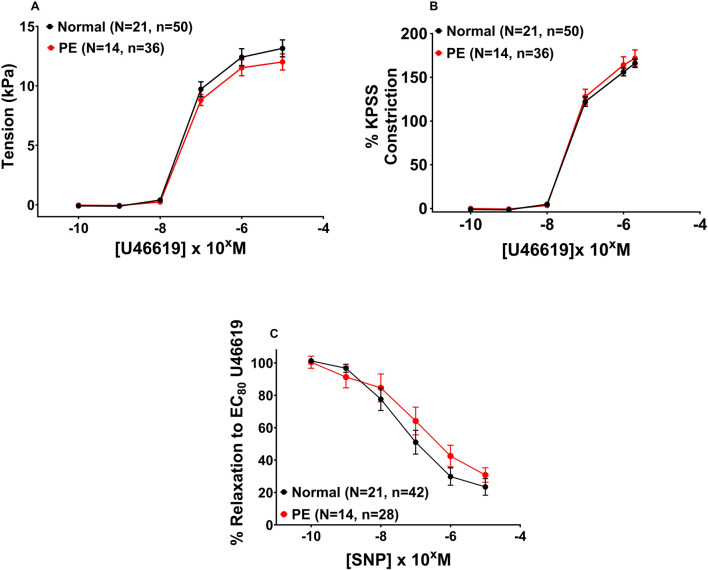
Assessment of vascular reactivity in CPAs from normal and PE pregnancies. Assessment of U46619-induced contraction expressed as tension **(A)** or as %KPSS **(B)**. Relaxation to SNP **(C)**. There was no significant difference between CPAs from normal *versus* PE pregnancies. Graph A shows the combined pre-incubation U46619 data for normal pregnancy (N = 21) and PE (N = 14) from pitavastatin, pravastatin, and simvastatin experiments (at all concentrations), while Graph B shows post-incubation CPA data with control groups from PE and normal pregnancy. Replicate vessels from the same pregnancy were averaged for each group. Data are expressed as mean ± SEM, two-way ANOVA.

Figure 4 includes all vessels prior to exposure to statin or DMSO. The endothelial-independent SNP dose–response curves shown are for DMSO controls from normal pregnancies and DMSO controls from PE pregnancies (post-incubation). A SNP dose–response curve was not conducted in PSS alone (pre-incubation). Contraction to U46619 expressed as tension (kPa) (Figure 4A, p = 0.700) and %KPSS (Figure 4B, p = 0.930), as well as relaxation of chorionic plate arteries to SNP (Figure 4C, p = 0.623) was not different between CPAs from normal *versus* PE pregnancies. When DMSO and water controls were separated to assess any differences between them, there was no significant difference in CPA relaxation to SNP between DMSO and water controls from normal pregnancies (p = 0.814) or PE pregnancies (p = 0.909).

### 3.5 Chorionic plate arteries from normal pregnancies

#### 3.5.1 Effect of statins on vasoconstriction of chorionic plate arteries (1 μM and 10 µM)

**FIGURE 5:**
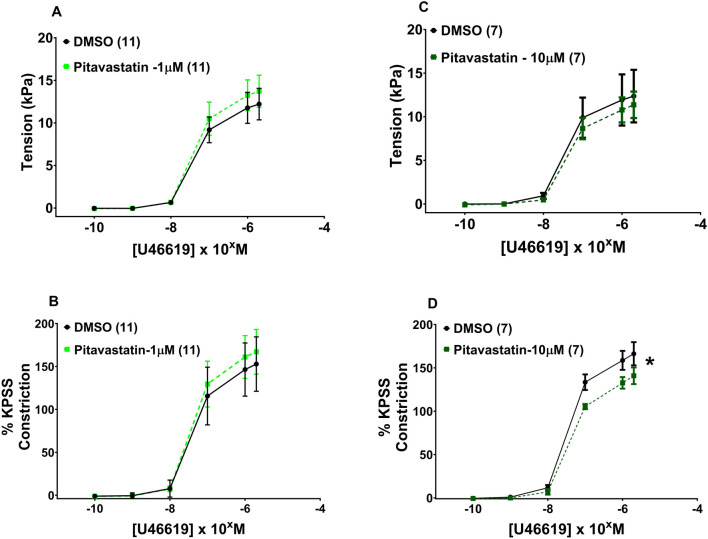
Effect of 2-h statin incubation on the contraction of CPAs from normal pregnancies. Assessment of U46619-induced contraction with either 1 µM pitavastatin **(A, B)** or 10 µM pitavastatin **(C)**. There was no significant difference between the statin-exposed and control groups. Only when expressed as % KPSS was a significant decrease in contraction seen with 10 µM pitavastatin **(D)**; p < 0.05. U46619 dose–response curves were compared using two-way ANOVA. Data are expressed as mean ± SEM; number of placentas in parentheses.

Following 2-h incubation with either 1 µM pitavastatin **(** Figure 5A, p = 0.924**)** or 10 µM pitavastatin **(** Figure 5C, p = 0.990), CPA contraction to U46619 was not significantly different between the pitavastatin-exposed group *versus* controls. When U46619 contraction curves were expressed as %KPSS with 1 µM pitavastatin **(** Figure 5B, p = 0.912), there was again no significant difference between pitavastatin-exposed CPAs and controls, but 10 µM pitavastatin attenuated the contraction of CPAs **(** Figure 5D, p = 0.026**)**. There was no difference in contraction to U46619 in the pravastatin and simvastatin groups (Supplementary Figure S4).

#### 3.5.2 Effect of statins on vasodilatation of chorionic plate arteries (1 μM and 10 µM)

**FIGURE 6:**
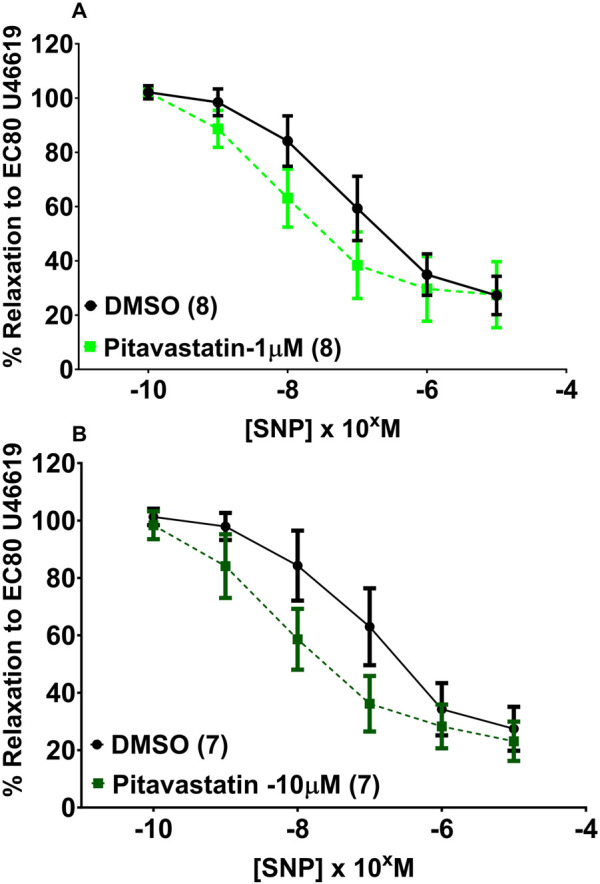
Effect of 2-h pitavastatin incubation on relaxation of CPAs from normal pregnancies. Assessment of CPA relaxation to SNP with either pitavastatin at 1 µM **(A)** or 10 µM pitavastatin **(B)**. No significant effect was seen between the statin-exposed and control groups; p > 0.05. SNP dose–response curves were compared using two-way ANOVA with Sidak’s *post hoc* test, where applicable. Data are expressed as mean ± SEM; number of placentas in parentheses.

Following a 2-h incubation with either 1 µM pitavastatin (Figure 6A, p = 0.086) or 10 µM pitavastatin **(** Figure 6B, p = 0.147), overall CPA relaxation to SNP was not significantly different between the pitavastatin-exposed group *versus* controls (Figure 6A, p = 0.086), although a trend was seen with 1 µM pitavastatin (p = 0.086). There was no difference in vasodilation to SNP in the pravastatin and simvastatin groups (Supplementary Figure S5).

### 3.6 Chorionic plate arteries from PE pregnancies

#### 3.6.1 Effect of statins on vasoconstriction of chorionic plate arteries in PE (1 μM and 10 µM)

**FIGURE 7:**
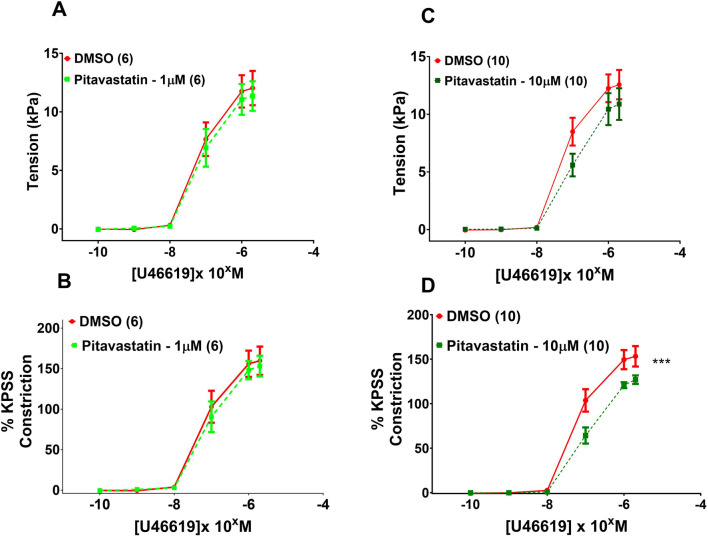
Effect of 2-h statin incubation on contraction of CPAs from PE pregnancies. Assessment of U46619-induced contraction with either 1 µM pitavastatin **(A,B)** or 10 µM pitavastatin **(C)**. There was no significant effect when expressed as tension (kPa) or as % KPSS; p > 0.05. However, a significant effect was seen for 10 µM pitavastatin **(D)** when data were expressed as % KPSS; p < 0.05. U46619 dose–response curves were compared using two-way ANOVA with Sidak’s post hoc test, where applicable. Data are expressed as mean ± SEM; number of placentas in parentheses.***p < 0.001.

Following a 2-h incubation with 1 µM pitavastatin (Figure 7A, p = 0.971**)** and 10 µM pitavastatin (Figure 7C, p = 0.245), CPA contraction to U46619 was not significantly different between the pitavastatin-exposed group *versus* controls. When U46619 contraction curves were expressed as %KPSS, with 1 µM pitavastatin **(** Figure 7B, p = 0.891), CPA contraction to U46619 was not significantly different between the pitavastatin-exposed group *versus* controls. However, with 10 µM pitavastatin, there was a decreased contractile response (Figure 7D, p = 0.0003). There was no difference in vasoconstriction to U46619 in the pravastatin and simvastatin groups (Supplementary Figure S8).

#### 3.6.2 Effect of statins on vasodilatation of chorionic plate arteries in PE (1 µM)

**FIGURE 8:**
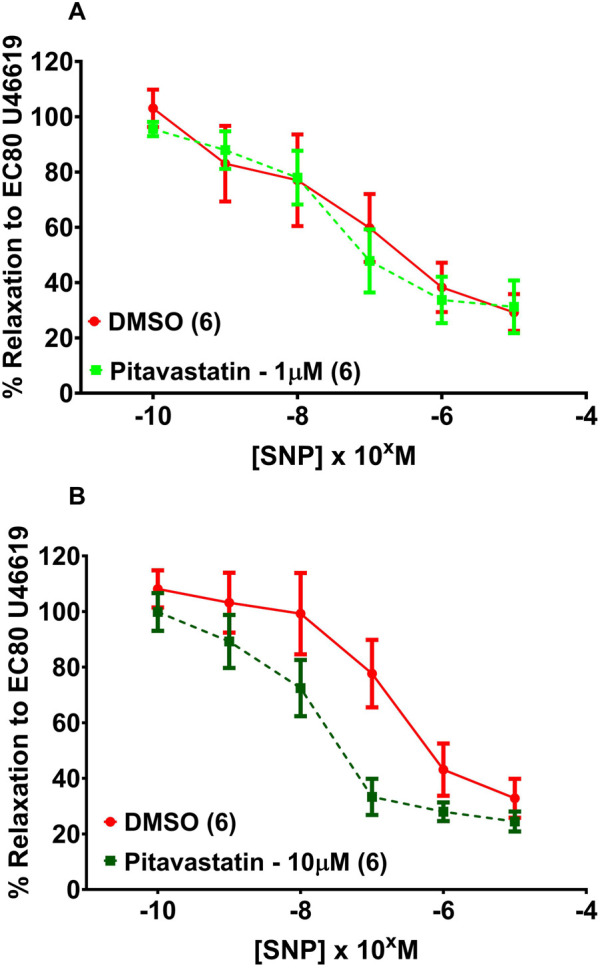
Effect of 2 hr statin incubation on relaxation of CPAs from PE pregnancies. Assessment of CPA relaxation to SNP with either 1 µM pitavastatin **(A)** or 10 µM pitavastatin **(B)**, there was no significant effect on relaxation between statin-exposed and control groups; p > 0.05. SNP dose response curves were compared using two-way ANOVA. Data are expressed as mean ± SEM; number of placentas in parenthesis.

Following a 2-h incubation with either 1 µM pitavastatin (Figure 8A, p = 0.961) or 10 µM pitavastatin (Figure 8B, p = 0.195), CPA relaxation to SNP was not significantly different between the pitavastatin-exposed group *versus* controls. There was no difference in CPA relaxation to SNP in the pravastatin and simvastatin groups (Supplementary Figure S9).

## 4 Discussion

The current study demonstrated evidence of endothelial dysfunction in OAs from pregnancies with PE, but short-term pitavastatin exposure (at 1 and 10 µM) had no effect on vascular reactivity in these vessels. Although there was no difference in vasorelaxation or vasoconstriction in CPAs between PE and normal pregnancies, there was no evidence of statins having detrimental effects on vascular reactivity in CPAs, and in fact, 10 µM pitavastatin attenuated U46619-mediated contraction in both PE and normal pregnancies. Whilst these data suggest that statins do not show any deleterious effects on the fetoplacental vasculature, the data also do not support pitavastatin being a potential drug treatment to improve maternal vascular function in preeclampsia.

### 4.1 Vascular reactivity of omental arteries in normal and PE pregnancies

OAs from women with PE did not show increased U46619-induced contraction compared to OAs from normotensive women**.** Multiple studies using different vasoconstrictive agents have shown that OAs from PE women showed increased contractile response compared to normotensive women (Aalkjaer et al., 1985; Mishra et al., 2011; Pascoal et al., 1998; Wareing et al., 2006b). Like the data in the current study, other studies have demonstrated no difference in contraction compared to normotensive women (Belfort et al., 1996; Knock and Poston, 1996; Vedernikov et al., 1995; Walsh, 1985; Wolff et al., 1996; Vedernikov et al., 1999). In the present study, thromboxane receptor expression, agonist affinity, and signal transduction post-receptor activation may not have been enhanced, resulting in no difference in contraction between normotensive and PE OAs. The selected testing method could also contribute to differences as some studies used pressure myography, while the current study used wire myography. Pre-incubation, endothelial function appeared to be significantly reduced in OAs from PE pregnancies compared to normotensive pregnancies following a single dose of bradykinin. However, because a control BK relaxation curve was not performed with PSS, we could not demonstrate differences in endothelium-dependent relaxation at different BK doses. The pitavastatin control, DMSO, at a dose of 0.1%, was able to cause maximal relaxation of vessels from normotensive and PE pregnancies, potentially masking endothelial dysfunction. However, the numbers are relatively small to be certain of this outcome. Other studies in different vascular beds have demonstrated attenuated endothelium-dependent relaxation to different vasodilatory agents in vessels from hypertensive pregnancies compared to normotensive pregnancies (Ashworth et al., 1997; Knock and Poston, 1996; Kublickiene et al., 1997; Mccarthy et al., 1994a).

### 4.2 Vascular reactivity of omental arteries following pitavastatin exposure in normal and PE pregnancies

Pitavastatin was used because it is a novel statin with high bioavailability in the bloodstream and has never been used in pregnancy studies. The doses used in this study are suprapharmacological *versus* those previously measured in human serum. For pitavastatin: 1 µM is 4× higher than a 4-mg dose in non-pregnant women, and 10 µM is 40× higher (Luo et al., 2015). Independent of their cholesterol-lowering ability, statins have been shown to increase the bioavailability of NO through mechanisms including, but not limited to, raised expression of eNOS and activation of the eNOS enzyme, as well as decreasing oxidative stress (Takemoto and Liao, 2001). In male Wistar rats, when the aorta and superior mesenteric arteries were pre-constricted with EC_80_ noradrenaline, the direct application of simvastatin produced relaxation in a concentration-dependent manner in both the presence and absence of a functional endothelium. These effects were attributed to the release of both NO and vasodilator products from cyclooxygenase by a mechanism sensitive to O_2_ scavengers, superoxide dismutase (Alvarez De Sotomayor et al., 2000). It was suggested that this relaxation is associated with both Ca^2+^ release from intracellular stores and blockade of extracellular Ca^2+^ entry (Alvarez De Sotomayor et al., 2001). Finally, Mukai et al. demonstrated that, following a 2 h incubation with the hydrophobic cerivastatin (1 μM), ACh-induced endothelium-dependent relaxation was enhanced in the rat aorta via the PI3 kinase/Akt pathway, and they also demonstrated endothelium-independent relaxation via Kv channel-mediated smooth muscle hyperpolarizations (dose-dependent: 1–100 μM). Direct application of fluvastatin (a moderately hydrophilic statin) was also used in this study and was able to stimulate relaxation of vascular smooth muscle in the aorta and mesenteric arteries in a dose-dependent manner (1–300 μM); vessels were pre-constricted with prostaglandin F2 alpha (Mukai et al., 2003).

The present study did not show an additional effect of pitavastatin on contraction or relaxation in OAs from normal or pathological pregnancies. An important issue to highlight is that OAs from the pathological pregnancies achieved a relaxation equivalent to vessels from normotensive pregnancies, and therefore, there was no apparent pathology to correct. The use of DMSO may have contributed to this, which could have altered the endothelial function of OAs post-incubation. Furthermore, different doses of statins were used in other studies, and there were differences in experimental design, as direct application of statins in the form of a dose-response curve was not performed in the present study. The use of different statin concentrations in the studies on omental arteries, as per studies in chorionic plate arteries, would have been insightful. Additionally, the current study focused upon resistance vessels in a closed system in the absence of flow, and hence, 2 h may not have been sufficient time to observe an effect on the endothelium. It is also possible that some of the statin pleiotropic effects, such as eNOS activation or alleviation of inflammation or oxidative stress, failed to occur within the timeline of these studies. Clinically, in a high-risk subpopulation, women may be given statins prophylactically during pregnancy; thus, maternal vessels may be exposed to statins despite the absence of disease. Thus, this study suggests that we did not see any detrimental effects of pitavastatin on the vascular reactivity of vessels from normal pregnancies. Clinically, this is an important finding, but care should be taken in extrapolating these findings to the clinic, given the *ex vivo* protocol and short-term statin exposure in this study.

### 4.3 Vascular reactivity of chorionic plate arteries in normal and PE pregnancies

In the limited *ex vivo* studies of statins on human placenta, statins reduced IGF-1-mediated trophoblast proliferation, leading to caution being expressed about their use in pregnancy, particularly in the first trimester (Forbes and Westwood, 2008; Forbes et al., 2015; Kenis et al., 2005). Additionally, Nanovskaya et al. carried out dual perfusion of a placental lobule with 50 ng/mL pravastatin (based upon achieved serum concentrations following a 40-mg dose) and showed that pravastatin was transferred to the fetal circulation. However, pravastatin was also transferred back to the maternal circulation, hypothesized to be via the action of the efflux transporters, MRP2 and BCRP, located on the microvillous membrane (MVM) of the transporting epithelium of the placenta, the syncytiotrophoblast (Nanovskaya et al., 2013), likely limiting the concentrations of statins reached within the fetal circulation.

In CPAs from normal pregnancy, contraction and endothelium-independent relaxation were not significantly different compared to PE. Functional studies have demonstrated conflicting results regarding the aberrant dysfunction of CPAs from women with PE compared to CPAs from normotensive women. Studies have shown no difference in contraction (Ong et al., 2002), reduced maximal contraction (Wareing and Baker, 2004), and significantly greater contraction when comparing normal and PE placentas (Benoit et al., 2007; Bertrand et al., 1993).

The data in this study do not support the current literature suggesting that CPAs from PE pregnancies are less responsive to SNP or show altered response to U46619. DMSO has been suggested to cause the endothelium to release NO to trigger cGMP production in the smooth muscle to promote relaxation and decrease Ca2+ sensitivity, partially through inhibiting Rho-kinase to attenuate constriction. However, we saw no evidence of this in the current study compared to water (vehicle). Other potential confounders that could have affected the results in the current study include the presence of smokers in the PE group, as smoking is associated with the production of carbon monoxide (CO) (Karumanchi and Levine, 2010; Wei et al., 2015). Furthermore, factors such as the effects of analgesia (Samanta et al., 2018) and the use of anti-coagulants (Atallah et al., 2017), such as aspirin and heparin, could alter vascular responses measured by wire myography.

### 4.4 Vascular reactivity of chorionic plate arteries following pitavastatin exposure in normal and PE pregnancies

The levels of vasodilators and vasoconstrictors in the maternal and placental circulation are regulated to ensure a homeostatic balance of placental vascular function. From a safety perspective, it is crucial that statins do not negatively affect this differential vascular response of the placenta as this could affect any adaptive responses the placenta may try to initiate in response to stimuli or stresses (Myatt, 1992; Wang and Zhao, 2010). Balan et al., who utilized perfused normal placental cotyledons and placental explants, demonstrated that feto-placental vascular tone or vasoconstriction in response to the vasoconstrictor angiotensin-II was unaltered following exposure to pravastatin from the maternal circulation (Balan et al., 2017). A pilot randomized controlled trial investigating the safety and pharmacokinetics of 10 mg pravastatin in high-risk PE women also showed no difference in infant birthweight between the pravastatin and placebo groups (Costantine et al., 2016).

The current study failed to demonstrate a beneficial effect of statins on the relaxation of CPAs from normal or PE pregnancies; however, 10 µM pitavastatin was able to attenuate U46619 contraction in CPAs from both normal and PE pregnancies. This suggests that pitavastatin may target the thromboxane A2 pathway to diminish its contractile effects, via antagonism of the thromboxane A2 receptor and/or thromboxane synthase in the endothelium (Kontogiorgis and Hadjipavlou-Litina, 2010). Alternatively, pitavastatin could diminish the anti-inflammatory effects of U46619 by blocking the ERK pathway and reducing expression of U46619-stimulated IL-1ß, IL-6, iNOS expression, and IL-1ß (Yang et al., 2016). Impaired vascular function in PE CPAs was not demonstrated, and the addition of statins did not have a biologically significant effect on vascular function; however, it was encouraging to see that no acute detrimental effect on vascular function occurred.

Limitations of the present study include the choice of statin concentration used in the *ex vivo* acute study. A suprapharmacological concentration was chosen because, for *ex vivo* myography experiments, a higher dose than those typically achieved *in vivo* is generally needed to result in similar effects. Furthermore, the endothelin-1 pathway was not explored directly. In addition, due to the relative lack of differences in the present study, extensive mechanistic studies were not performed, but future studies could include assessments of whether statins altered eNOS signaling/activation and levels of oxidative stress/damage. These may also help explain the lack of any effects of statins on omental artery function in the current study. Finally, it is important to acknowledge that several women in the PE group were prescribed aspirin and antihypertensive medication at various points within their pregnancy, and this may have contributed to altered vascular functional responses *ex vivo*.

## 5 Conclusion

Taking into account the data from this study using pravastatin, pitavastatin, and simvastatin, statins did not negatively affect vascular function in placental vessels, suggesting that these drugs are unlikely to have a large vasoactive effect within the placenta at physiological doses. The outcome of the *in vitro* data does not provide convincing evidence for pitavastatin as an optimal treatment to modify endothelial function in PE, but there are limitations to this study, such as the acute exposure time, as detailed above. Mechanistic studies, such as increased exploration of statins’ pleiotropic effects, are essential to providing a greater understanding of the potential clinical applications of statins in PE. This study focused on short-term effects of statins on vascular function but investigating the effects of longer-term statin exposure in pregnancy will be important to demonstrate its potential as a therapy in PE. This requires appropriate preclinical *in vivo* models to provide the necessary safety and mechanistic data to underpin further clinical trials. Overall, these data give greater confidence that, if statins are shown to be beneficial in the clinical treatment/management of PE, the doses used, at least in the short term, are unlikely to be detrimental to fetoplacental vascular function. While we failed to see a positive effect of pitavastatin on maternal omental artery function, this does not mean that other statins will not show beneficial effects. The future studies suggested above will provide improved mechanistic understanding of the validity of statin use in individuals with preeclampsia.

## Data Availability

The original contributions presented in the study are included in the article/Supplementary Material; further inquiries can be directed to the corresponding author.
