# Supplementary material for: Acute effect of statins on vascular reactivity in maternal and placental arteries from pregnancies complicated by preeclampsia
Source: Front Physiol. 2025 Jun 24;16:1575128. doi: 10.3389/fphys.2025.1575128 (PMC12234468; doi:10.3389/fphys.2025.1575128)
Supplement: Supplementary file 1 [file DataSheet1.docx]

# **Supplementary material**

**Data for omental samples only**

**Normal and PE pregnancies**

*Basal tone of omental small arteries*

Following a 2 hr incubation with pitavastatin (1 µM), the basal tone of OAs was not significantly different between the statin-exposed group versus controls in normal pregnancies ((Figure 1 A: DMSO vs pitavastatin (p=0.832)) or PE pregnancies (Figure 1 B: DMSO vs pitavastatin (p=0.297)). During experiments, we noticed that there was an effect of time on the vascular responsiveness of OAs, coupled with the effect of DMSO and pitavastatin on vasoactive mediators, this could have contributed to reduced basal tone over time.

**

**A**

**Normal pregnancies**

**B**

**PE pregnancies**

Figure 1: Effect of 2 hr incubation with 1 µM pitavastatin on basal tone of omental arteries relative to control group in normal and PE pregnancy

Assessment of the effect of 1 µM pitavastatin on basal tone, there was no significant difference between the statin-exposed group versus controls in normal pregnancies (A) or PE pregnancies (B). The horizontal line denotes median and data are expressed as median±IQR; Mann-Whitney U test performed.

**Data for placental samples only**

**Normal pregnancies**

*Basal tone of chorionic plate arteries (*1 µM and 10 µM)

Following a 2 hr incubation with either 1µM pitavastatin (Figure 2 A, p=0.898) or 10 µM pitavastatin (Figure 2 B, p=0.259) basal tone of CPAs was not significantly different between the pitavastatin-exposed group versus controls**.**

**A**


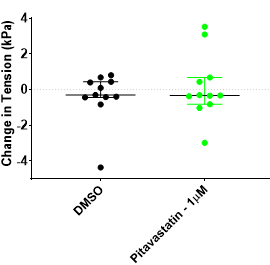

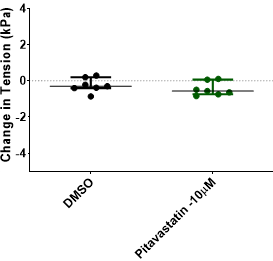


**B**

**Figure 2**: Effect of 2 hr incubation with statins on basal tone of chorionic plate arteries from normal pregnancies relative to control group.

Assessment of CPA basal tone with either 1 µM pitavastatin (A), or 10 µM pitavastatin (B), there was not a significant difference between statin-exposed group versus controls. The horizontal line denotes median and data expressed as median±IQR; Mann-Whitney U test performed.

Following a 2 hr incubation with either pravastatin (1 µM, Figure 3 A: **Water** vs **pravastatin (**p=0.529)), simvastatin (1 µM, Figure 3 B: **DMSO** vs **simvastatin (**p=0.336)), pravastatin (10 µM, Figure 3 C: **Water** vs **pravastatin** (p>0.9999)) basal tone of CPAs was not significantly different between statin-exposed group versus controls.

**B**

**A**

**Figure 3**: Effect of 2 hr incubation with statins on basal tone of chorionic plate arteries from normal pregnancies relative to control group.

Assessment of CPA basal tone with 1 µM pravastatin (A), 1 µM simvastatin (B), or 10 µM pravastatin (C), there was not a significant difference between statin-exposed group versus controls. The horizontal line denotes median and data expressed as median±IQR; Mann-Whitney U test performed.

**C**

**Contraction of chorionic plate arteries**

*Vasoconstriction of chorionic plate arteries (1 µM and 10 µM)*

Following a 2 hr incubation with 1 µM pravastatin (Figure 4 A: **Water** vs **pravastatin (**p=0.088)**),** with 10 µM pravastatin (Figure 4 B: **Water** vs **pravastatin (**p=0.998)**)** and simvastatin **(**Figure 4 C: **DMSO** vs **simvastatin (**p=0.778**)**) there was no significant effect on CPA contraction to U46619 from normal pregnancies. When U46619 contraction curves were expressed as %KPSS there was again no significant difference between statin-exposed CPAs and comparable control CPAs; 1 µM (Figure 4 D: **Water** vs **pravastatin (**p=0.974)**),** 10 µM (Figure 4 E: **Water** vs **pravastatin (**p=0.998**)),** and **(**Figure 4 F: **DMSO** vs **simvastatin (**p=0.973**)**).

**Figure 4:** Effect of 2 hr statin incubation on contraction of CPAs from normal pregnancies.

Assessment of U46619 induced contraction with either 1 µM pravastatin (A), 10 µM pravastatin (B) or 1 µM simvastatin (C), there was no significant difference between statin-exposed and control groups; p<0.05. U46619 dose response curves were compared using two-way ANOVA. Data are expressed as mean±SEM; number of placentas in parenthesis.

**A**

**B**

**C**

**F**

**E**

**D**

**Vasodilation**

*Effect of statins on vasodilatation of chorionic plate arteries (*1 µM and 10 µM)

Following a 2 hr incubation with 1 µM pravastatin (Figure 5 A: **Water** vs **pravastatin (**p=0.945**),** with 10 µM pravastatin (Figure 5 B: **Water** vs **pravastatin (**p=0.928**)**  and simvastatin **(**Figure 5 C: **DMSO** vs **simvastatin (**p=0.870**))** there was no significant effect on CPA relaxation to SNP from normal pregnancies.

**B**

**A**

**C**

**Figure 5**: Effect of 2 hr pitavastatin incubation on relaxation of CPAs from normal pregnancies.

Assessment of CPA relaxation to SNP with either 1 µM pravastatin (A), 10 µM pravastatin (B) or 1 µM simvastatin (C), there was no significant effect seen between statin-exposed and control group; p>0.05. SNP dose response curves were compared using two-way ANOVA with Sidak’s *post hoc* test, where applicable. Data are expressed as mean±SEM; number of placentas in parenthesis.

**Basal tone of chorionic plate arteries - PE Pregnancies**

*Effect of statins on basal tone of chorionic plate arteries (*1 µM and 10 µM)

Following a 2 hr incubation with either 1 µM pitavastatin (Figure 6 A, p=0.342) or 10 µM pitavastatin (Figure 6 B, p=0.143), basal tone of CPAs was not significantly different between statin-exposed group versus controls.

**A**

**Figure 6**: Effect of 2 hr incubation with statins on basal tone of chorionic plate arteries from PE pregnancies relative to control group.

Assessment of basal tone following a 2 hr incubation with 1 µM pitavastatin at (A) or 10 µM pitavastatin (B), there was no significant difference between statin-exposed group versus controls. The horizontal line denotes median, and data expressed as median±IQR; Mann-Whitney U test performed.

**B**

*Basal tone of chorionic plate arteries (*1µM)

Following a 2 hr incubation with either pravastatin (1 µM, Figure 7 A: **Water** vs **pravastatin (**(p=0.517)) or simvastatin (1 µM, Figure 7 B: **DMSO** vs **simvastatin (**(p=0.383)) basal tone of CPAs was not significantly different between statin-exposed group versus controls.

**A**

**B**

**Figure 7**: Effect of 2 hr incubation with statins on basal tone of chorionic plate arteries from PE pregnancies relative to control group.

Assessment of basal tone following a 2 hr incubation with 1 µM pravastatin (A) or simvastatin (B), there was no significant difference between statin-exposed group versus controls. The horizontal line denotes median, and data expressed as median±IQR; Mann-Whitney U test performed.

**Contraction of chorionic plate arteries - PE Pregnancies**

*Vasoconstriction of chorionic plate arteries (1 µM)*

Following a 2 hr incubation with pravastatin (Figure 8 A: **Water** vs **pravastatin (**p=0.561)**),** and simvastatin **(**Figure 8 B: **DMSO** vs **simvastatin (**(p=0.387**)**), there was no significant effect on CPA contraction to U46619 from PE pregnancies. When U46619 contraction curves were expressed as %KPSS there was again no significant difference between statin-exposed CPAs and comparable control CPAs; (Figure 8 C: **Water** vs **pravastatin (**p=0.535)**),** and **(**Figure 8 D: **DMSO** vs **simvastatin (**p=0.785**)**).

**B**

**A**

**D**

**C**

**Figure 8:** Effect of 2 hr statin incubation on contraction of CPAs from PE pregnancies.

Assessment of U46619-induced contraction with either 1 µM pravastatin (A, C) or 1 µM simvastatin (B, D), there was no significant effect when expressed as tension (kPa) or as % KPSS; p>0.05. U46619 dose response curves were compared using two-way ANOVA with Sidak’s post hoc test, where applicable. Data are expressed as mean±SEM; number of placentas in parenthesis. ***P<0.001.

**Vasodilation of chorionic plate arteries - PE Pregnancies**

*Vasodilation of chorionic plate arteries (1 µM)*

Following a 2 hr incubation with pravastatin (Figure 9 A: **Water** vs **pravastatin** (p=0.913), and simvastatin (Figure 9 B: **DMSO** vs **simvastatin** (p=0.496)), there was no significant effect on CPA relaxation to SNP from pregnancies with PE.

**B**

**A**

**Figure 9:** Effect of 2 hr statin incubation on relaxation of CPAs from PE pregnancies.

Following a 2 hr incubation with pravastatin, pitavastatin and simvastatin at 1 µM, there was no significant effect on relaxation of CPAs to SNP from PE pregnancies; p>0.05. SNP dose response curves were compared using two-way ANOVA. Data are expressed as mean±SEM; number of placentas in parenthesis.

**Chorionic plate arteries: flowchart of vessels used and excluded**

**
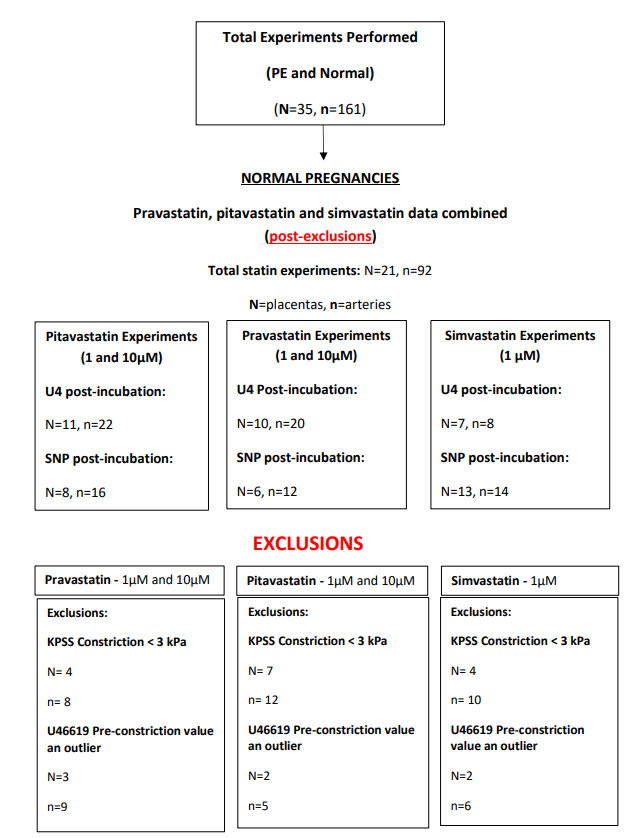
**

**
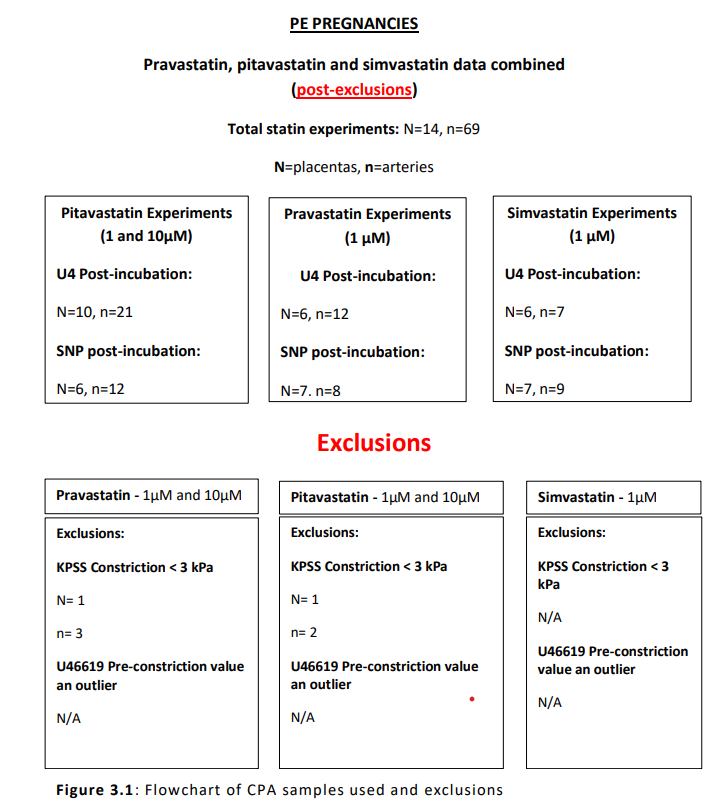
**

**Omental arteries: flowchart of vessels used and excluded**

**
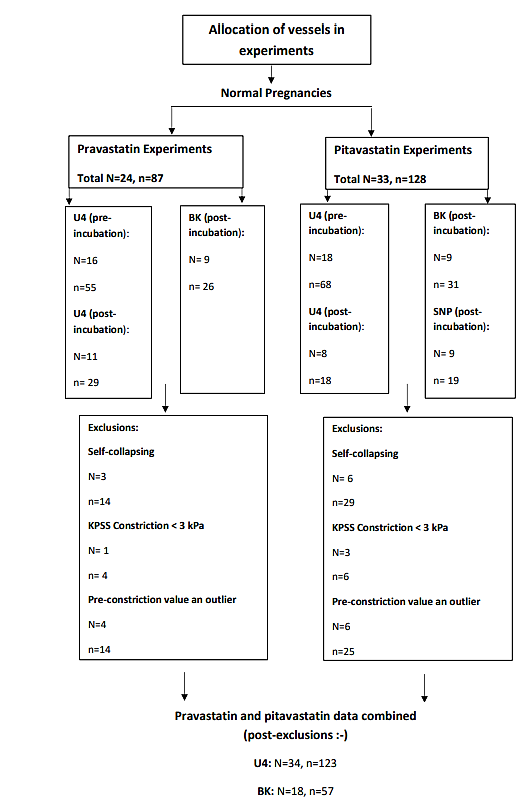
**
